# COVID-19 induces lower levels of IL-8, IL-10, and MCP-1 than other acute CRS-inducing diseases

**DOI:** 10.1073/pnas.2102960118

**Published:** 2021-05-10

**Authors:** Liang Cheng, Zijun Zhu, Chao Wang, Ping Wang, Yongqun Oliver He, Xue Zhang

**Affiliations:** ^a^NHC and CAMS Key Laboratory of Molecular Probe and Targeted Theranostics, Harbin Medical University, Heilongjiang 150028, China;; ^b^College of Bioinformatics Science and Technology, Harbin Medical University, Heilongjiang 150081, China;; ^c^Department of Microbiology and Immunology, University of Michigan Medical School, Ann Arbor, MI 48109;; ^d^McKusick-Zhang Center for Genetic Medicine, Peking Union Medical College, Beijing 100005, China

Kang et al. (1) investigate the relations between the cytokine release syndrome (CRS), cytokines, and different acute diseases that cause CRS. Their study finds strikingly elevated levels of four proinflammatory cytokines, including IL-6, IL-8, IL-10, and MCP-1, in 91 patients with CRS associated with sepsis, acute respiratory distress syndrome (ARDS), or burns. Furthermore, the elevated levels of these cytokines are positively correlated with the increasing expression of the coagulation cascade activator plasminogen activator inhibitor-1 (PAI-1). PAI-1 is capable of causing small blood clots in blood vessels throughout the body (including pulmonary blood vessels). Importantly, the increase of PAI-1 is associated with more severe pneumonia, a common cause of death in COVID-19 patients. Finally, they find that the treatment with tocilizumab, a human monoclonal antibody that blocks IL-6 signal transduction, decreases the PAI-1 levels and alleviates severe respiratory complications in COVID-19 patients. We agree with their views on the function of IL-6 and PAI-1 in CRS. Kang et al. (1) also observe an important clinical characteristic: that the cytokines IL-6, IL-8, IL-10, and MCP-1, in the plasma of severe COVID-19 patients, are not as high as those in CRS patients with sepsis, ARDS, and burns. However, the authors did not perform statistical tests to directly investigate the associations between COVID-19 and these cytokines, which prompted us to statistically test their observations.

Instrumental variables (IVs) were introduced to explore the changing of the cytokines in COVID-19 using a two-sample Mendelian randomization (MR) analysis (2), a statistical method that has been applied for identifying the risk factors of COVID-19 (3). IVs were screened from two genome-wide association studies (GWAS): one GWAS of severe COVID-19 with respiratory failure (1,610 severe patients and 2,205 healthy controls in Italy and Spain) (4) and the other GWAS of circulating cytokines (8,293 Finnish individuals) (5). Single nucleotide polymorphisms that could be regarded as IVs must meet three standards: 1) significantly related to COVID-19 (*P* value < 1.0 × 10^−5^); 2) nonlinkage disequilibrium (*R*^2^ < 0.001); and 3) no association with cytokines (*P* value > 0.05). Using methods previously reported, the inverse-variance weighted (IVW) method and MR-Egger regression were conducted for main MR analysis and horizontal pleiotropy evaluation of IVs, respectively (6, 7). Based on the IVW analysis, the levels of IL-8, IL-10, and MCP-1 in COVID-19 are found to be significantly lowered in patients with CRS (Table 1). The low intercepts obtained from MR-Egger regression further indicate that the horizontal pleiotropy is unlikely to cause a bias in the levels of IL-8 and MCP-1, and IVs are unlikely to affect the level of IL-10.

**Table 1.** MR analysis of the causal effect of COVID-19 on IL-6, IL-8, IL-10, and MCP-1

Cytokines (the number of IVs)	Odds ratio based on IVW	95% CIs based on IVW	*P* value based on IVW	Intercept based on MR-Egger (*P* value)
IL-6 (113)	0.9910	(0.9820, 1.0001)	0.0527	−0.0325 (9.64E-05)
IL-8 (116)	0.9765	(0.9632, 0.9899)	0.0007	−0.0225 (0.0582)
IL-10 (115)	0.9859	(0.9767, 0.9952)	0.0029	0.0173 (0.0332)
MCP-1 (32)	0.9641	(0.9469, 0.9815)	6.4E-05	0.0046 (0.6743)

Overall, our MR analysis shows that COVID-19 induced lower levels of IL-8, IL-10, and MCP-1 than other acute diseases in CRS patients, suggesting a critical COVID-19 pathogenesis mechanism, that is, lowering the levels of these three cytokines in COVID-19 patients with CRS compared with the levels in those patents with other acute CRS-inducing diseases including bacterial sepsis, ARDS, and burns.
